# Supplementary material for: A2A Receptor Dysregulation in Dystonia DYT1 Knock-Out Mice
Source: Int J Mol Sci. 2021 Mar 7;22(5):2691. doi: 10.3390/ijms22052691 (PMC7962104; doi:10.3390/ijms22052691)
Supplement: Supplementary file 1 [file ijms-22-02691-s001.pdf]

Supplementary Material

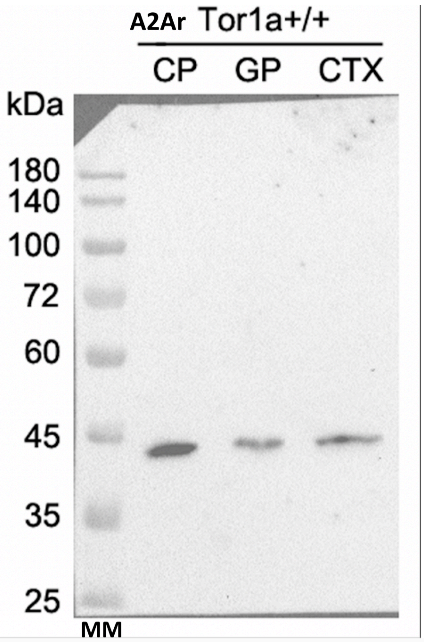

Figure 1. Image of Western blot analysis of A2A receptors in tissue homogenates of the caudate-putamen (CP), globus pallidus (GP), and cerebral cortex (CTX). Reference of molecular weight markers (MM) on the left column. A specific A2A receptor band at about 44-45 kDa is detected in the three brain areas, more intense in the CP.
